# Engagement and retention with remote monitoring in psoriasis: Findings from the mySkin study

**DOI:** 10.1111/jdv.70153

**Published:** 2025-10-29

**Authors:** Weiyu Ye, Niamh Dooley, Wei Ren Tan, Bolaji Coker, Teresa Tsakok, Lucy Moorhead, Jade Pizzato, Kingsley Powell, Madeline Kroah‐Hartman, Alexandra Paolino, Treasa Jiang, Freya Jackson‐Duffy, John Y. W. Lee, Manpreet K. Sagoo, Georgia Sewell, Camille Lancelot, Helen McAteer, John Weinman, Jonathan N. Barker, Sarah Chapman, Sam Norton, Catherine H. Smith, Satveer K. Mahil

**Affiliations:** ^1^ St John's Institute of Dermatology, King's College London and Guy's and St Thomas' NHS Foundation Trust London UK; ^2^ Social, Genetic and Developmental Psychiatry Centre King's College London London UK; ^3^ Research and Development Guy's and St Thomas' NHS Foundation Trust London UK; ^4^ University of Groningen Groningen The Netherlands; ^5^ Psoriasis Association Northampton UK; ^6^ International Federation of Psoriasis Association Stockholm Sweden; ^7^ Centre for Adherence Research and Education King's College London London UK; ^8^ Centre for Rheumatic Diseases King's College London London UK

**Keywords:** patient‐reported outcomes, photograph, psoriasis, remote monitoring, retention

Dear Editor,

Longitudinal remote monitoring enables real‐time tracking of outcomes in individuals with long‐term immune‐mediated inflammatory conditions, with the potential to inform personalized healthcare and enhance research data collection efficiency. In dermatology, self‐taken photographs provide objective information complementary to patient‐reported outcomes. As willingness and ability to engage with remote monitoring may vary,^1^ identifying factors influencing participation is essential for equitable care delivery.

We examined demographic/clinical factors associated with engagement and retention with a remote monitoring platform in mySkin, a longitudinal UK‐wide cohort study of adults with psoriasis investigating environmental determinants of disease onset and worsening.^2^ Participants self‐enrolled via myskin.org (17 June 2023–present) and completed 3‐monthly surveys for 12 months, with the option to submit self‐taken photographs. Demographic/clinical data and self‐reported triggers to psoriasis onset and worsening were collected.^2^ Uni/multivariate analyses identified factors influencing participation, with candidate factors based on prior research^3^, ^4^ and study team consensus.

Of the 864 individuals completing ≥1 survey (data extracted 26 November 2024), most were of White ethnicity (89.9%) and female (62.3%) (Table 1). At baseline, 53.0% agreed to submit optional photographs. Agreement was more likely among younger individuals (*p* = 0.01), those with shorter psoriasis duration (*p* < 0.001), higher self‐reported severity (*p* < 0.001) and no psoriatic arthritis (*p* = 0.04). In multivariate analysis, self‐reported severity remained significant (OR 2.66, 95% CI 1.81–3.90). Of those who agreed, 41.7% submitted photographs. Submission was more likely among unemployed individuals (*p* = 0.01) and those with non‐facial psoriasis (*p* = 0.03).

**TABLE 1:** mySkin study population characteristics at baseline.

Demographic and clinical characteristics	
Sex, *n* (%)	
Female	538 (62.3)
Male	325 (37.6)
Prefer not to say	1 (0.1)
Age, mean (SD)	51.0 (14.1)
Ethnicity, *n* (%)	
White	777 (89.9)
Non‐White^a^	87 (10.1)
Employment status, *n* (%)	
Employed	575 (66.6)
Unemployed (retired)	165 (19.1)
Unemployed (disability)	53 (6.1)
Unemployed (other^b^)	71 (8.2)
Index of multiple deprivation quintile, *n* (%)	
1 (most deprived)	74 (8.6)
2	134 (15.5)
3	162 (18.8)
4	175 (20.3)
5 (least deprived)	152 (17.6)
Missing responses	167 (19.3)
Psoriasis duration (years), mean (SD)	28.3 (16.9)
Family history of psoriasis, *n* (%)	421 (48.7)
Self‐assessed psoriasis severity (PtGA), *n* (%)	
Clear or nearly clear	257 (29.7)
Mild	226 (26.2)
Moderate to severe	381 (44.1)
Psoriasis affecting high‐impact sites, *n* (%)^c^	
Scalp	503 (62.1)
Nail	310 (38.3)
Palm/soles	82 (10.1)
Flexural	162 (20.0)
Face	211 (26.0)
Psoriasis treatment, *n* (%)	
None	53 (6.13)
Topical treatment	591 (68.4)
Targeted biologic therapy^d^	280 (32.4)
Conventional systemic immunomodulator^e^	154 (17.8)
Phototherapy	29 (3.4)
Psoriatic arthritis, *n* (%)	244 (28.2)
Mental health comorbidity^f^, *n* (%)	228 (26.4)
Number of physical health comorbidities, mean (SD)	1.29 (1.39)

*Note*: Data for 864 participants.

Abbreviations: PtGA, Patient Global Assessment, a 6‐point Likert scale (clear, nearly clear, mild, moderate, moderate–severe, severe). In the analysis, we combined clear and nearly clear categories, and moderate, moderate–severe and severe categories; SD, standard deviation.

^a^ Non‐White ethnicity categories include Mixed, Asian, Black, Arab and Other ethnicity.

^b^ Unemployed (other) categories include unpaid/voluntary work, student, carer and other.

^c^ Participants can have psoriasis affecting multiple high‐impact sites.

^d^ Targeted biologic therapy includes adalimumab, brodalumab, bimekizumab, certolizumab, etanercept, golimumab, guselkumab, infliximab, ixekizumab, risankizumab, secukinumab, tildrakizumab and ustekinumab.

^e^ Conventional systemic immunomodulators include acitretin, apremilast, ciclosporin, fumaric acid esters and methotrexate.

^f^ Mental health comorbidities include depression, anxiety, bipolar disorder, schizophrenia, personality disorder, eating disorder and others.

Retention at 3, 6, 9 and 12 months was 62.9%, 58.4%, 52.7% and 46.3%, respectively (Figure 1). Older age (OR 1.10, 95% CI 1.07–1.12), unemployment (OR 2.03, 95% CI 1.19–3.46; primarily due to retirement) and self‐reported mental health comorbidities (OR 2.01, 95% CI 1.20–3.37) were associated with higher retention. Participants with self‐reported clear/nearly clear skin (OR 2.66, 95% CI 1.58–4.48) or mild psoriasis (OR 2.29, 95% CI 1.33–3.94) had higher retention than those with moderate/moderate–severe/severe psoriasis.

**FIGURE 1:**
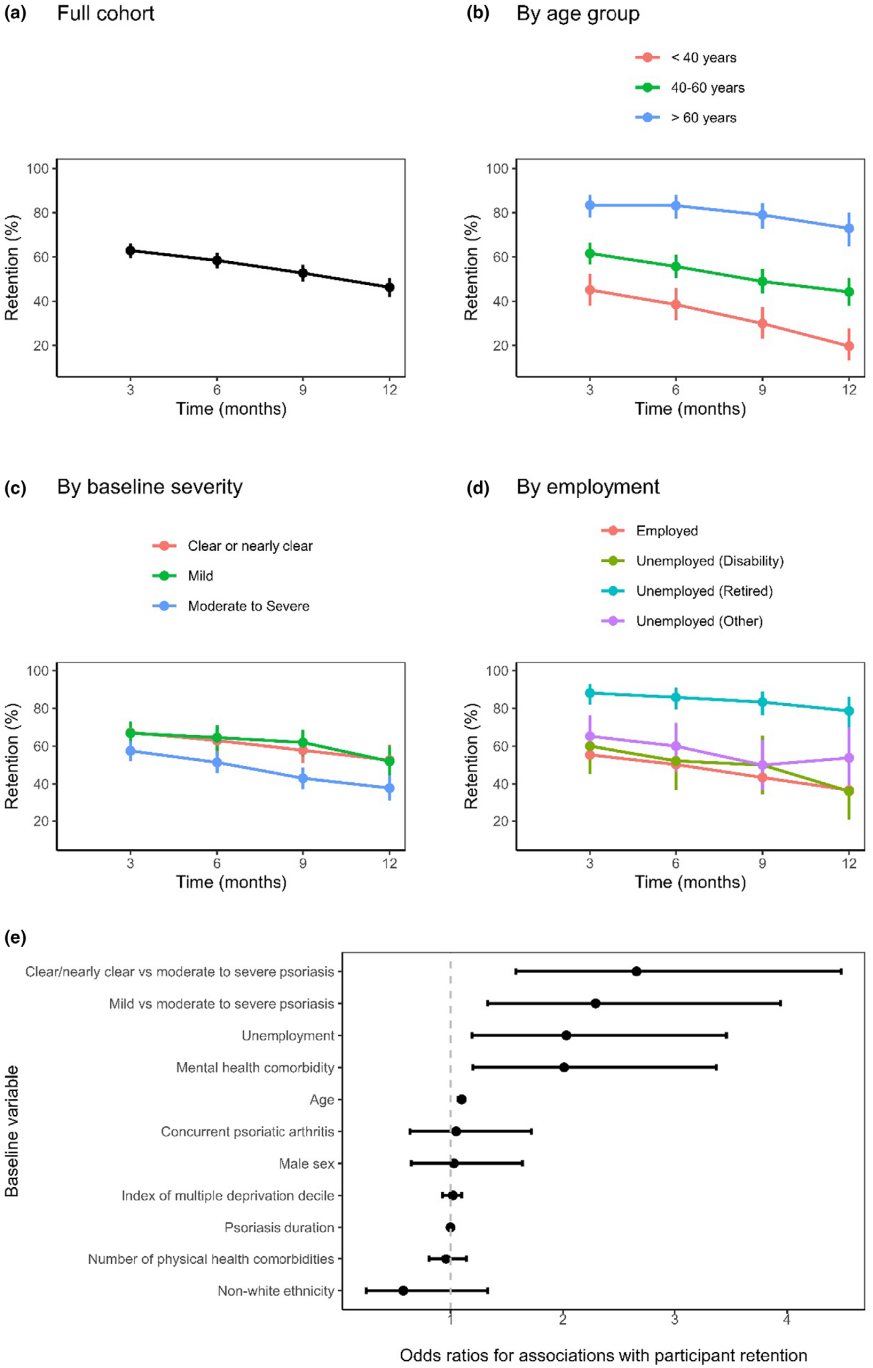
Participant retention in the mySkin longitudinal cohort study. (a) Overall retention for the full cohort and (b–d) retention stratified by subgroups. (e) Odds ratios for the associations between baseline demographic and clinical variables and participant retention, estimated using a logistic mixed model. Error bars denote 95% confidence intervals.

Our findings highlight variation in engagement with remote monitoring across patient groups. Consistent with previous studies, older individuals^5^, ^6^, ^7^ and those with well‐controlled disease^6^ showed higher retention. As more individuals achieve clear/nearly clear skin with effective biologic therapies, remote monitoring could reduce their healthcare burden while releasing clinical capacity for those in need. In inflammatory arthritis, a systematic review found remote monitoring reduced face‐to‐face visits.^8^ Prior psoriasis research has shown that teledermatology can achieve clinical outcomes equivalent to in‐person care, supporting its integration into real‐world practice.^9^

Our data indicate that younger, employed patients with severe psoriasis are more likely to disengage over time. While not harder to reach initially, they may require tailored support to sustain participation, such as clinician‐supported onboarding and personalized feedback to enhance clinician–patient communication.^3^, ^5^ Advances in artificial intelligence offer additional opportunities, with potential to monitor engagement patterns, detect early disengagement and deliver targeted support to improve retention.^10^ In research settings, financial incentives may boost engagement.^5^ Our findings underscore a need to identify barriers to photograph submission, which may include time constraints, technological issues and digital literacy. Stigma and privacy concerns may be important, as submission rates were higher among individuals with non‐facial psoriasis. Interviews with engaged/disengaged participants could yield deeper insights into patient behaviours.

Strengths of this study include its large sample size, UK‐wide recruitment and longitudinal design. Limitations include the predominance of female participants, underrepresentation from ethnic minority groups and English‐only surveys. Ascertainment of clinician perspectives will also be an important future focus. As a research study, findings may not fully generalize to real‐world clinical practice.

Digital transformation in healthcare relies on patient engagement and retention. Our study of longitudinal remote monitoring in psoriasis demonstrates variation in retention across patient groups, highlighting the need to tailor digital tools to optimise engagement with hard to reach and underserved groups, supporting equitable real‐world implementation.

## FUNDING INFORMATION

W.Y. is an NIHR Doctoral Training Fellow (NIHR304617). S.K.M. is supported by an NIHR Advanced Fellowship (NIHR302258) and C.H.S. by an NIHR Senior Investigator Award. Support for this work was also received from the Psoriasis Association (RE18933).

## CONFLICT OF INTEREST STATEMENT

C.H.S. reports departmental research funding from AstraZeneca, Boehringer Ingelheim, UCB and Sanofi outside the submitted work, and serves as an investigator in EU‐IMI consortia involving multiple industry partners (see biomap‐imi.eu and hippocrates‐imi.eu for details). S.K.M. reports departmental income from AbbVie, Almirall, Eli Lilly, Leo, Novartis, Sanofi and UCB outside the submitted work. L.M. has received consulting fees, speaker fees or support for attending meetings or travel from AbbVie, Alliance, Sanofi, Almirall, BMS, Dermal, Johnson & Johnson, Leo, Eli Lilly, Novartis, UCB and Pfizer. J.N.B. has attended advisory boards and/or received consultancy fees and/or spoken at sponsored symposia, and/or received grant funding from AbbVie, Amgen, Boehringer Ingelheim, Bristol‐Meyers‐Squibb, Johnson & Johnson, Lilly, Novartis and UCB. The remaining authors declare no competing interests.

## ETHICAL APPROVAL

The mySkin study has received approval from the HRA and REC (reference number 22/NI/0193).

## ETHICS STATEMENT

All patients provided written informed consent in accordance with the Declaration of Helsinki.

## Data Availability

The data underlying this article cannot be shared publicly due to linkage with other health resources as per current contracts. De‐identified data are potentially available subject to request.
